# Prevalence of Cancer

**Published:** 1882-08-20

**Authors:** 


### *PREVALENCE OF CANCER.*

The death of Senator HILL, of Georgia, which took place on the 16th inst., suggests to our minds the increased prevalence of cancerous affections in this country and the humilliating fact that we have yet no remedy for the disease. Another fact may as well be confessed by the profession, and that is, that the knife which has been claimed as the best method for removing cancerous tumors is, after all, a very questionable method, even in the milder cases where life is expected to be prolonged by removing the tumor, as experience shows that it more frequently hastens the fatal result either by enlarging the surface upon which the diseased process is renewed, or by causing the development of the malady in some internal or more vital part of the system.

